# Microenvironment inflammatory infiltrate drives growth speed and outcome of hepatocellular carcinoma: a prospective clinical study

**DOI:** 10.1038/cddis.2017.395

**Published:** 2017-08-24

**Authors:** Rosina Critelli, Fabiola Milosa, Francesca Faillaci, Rosario Condello, Elena Turola, Luca Marzi, Barbara Lei, Francesco Dituri, Silvia Andreani, Pamela Sighinolfi, Paola Manni, Antonino Maiorana, Cristian Caporali, Fabrizio di Benedetto, Mariagrazia Del Buono, Nicola De Maria, Filippo Schepis, Maria-Luz Martinez-Chantar, Gianluigi Giannelli, Erica Villa

**Affiliations:** 1 Department of Internal Medicine, Gastroenterology Unit, University of Modena and Reggio Emilia, Modena, Italy; 2 WomenInHepatology Network, Modena, Italy; 3 National Institute of Gastroenterology, “S. de Bellis” Research Hospital, Castellana Grotte; 4 Department of Pathology, University of Modena and Reggio Emilia, Modena, Italy; 5 Department of Radiology, University of Modena and Reggio Emilia, Modena, Italy; 6 Liver and Multivisceral Transplant Center, University of Modena and Reggio Emilia, Modena, Italy; 7 CIC bioGUNE, Centro de Investigación Cooperativa en Biociencias, Technology Park of Bizkaia, Derio, Bizkaia, Spain; 8 Centro de Investigación Biomédica en Red de Enfermedades Hepáticas y Digestivas (CIBERehd), Instituto de Salud Carlos III, Madrid, Spain

## Abstract

In HCC, tumor microenvironment, heavily influenced by the underlying chronic liver disease, etiology and stage of the tissue damage, affects tumor progression and determines the high heterogeneity of the tumor. Aim of this study was to identify the circulating and tissue components of the microenvironment immune-mediated response affecting the aggressiveness and the ensuing clinical outcome. We analyzed the baseline paired HCC and the surrounding tissue biopsies from a prospective cohort of 132 patients at the first diagnosis of HCC for immunolocalization of PD-1/PD-L1, FoxP3, E-cadherin, CLEC2 and for a panel of 82 microRNA associated with regulation of angiogenesis, cell proliferation, cell signaling, immune control and autophagy. Original microarray data were also explored. Serum samples were analyzed for a panel of 19 cytokines. Data were associated with biochemical data, histopathology and survival. Patients with a more aggressive disease and shorter survival, who we named fast-growing accordingly to the tumor doubling time, at presentation had significantly higher AFP levels, TGF-*β*1 and Cyphra 21-1 levels. Transcriptomic analysis evidenced a significant downregulation of CLEC2 and upregulation of several metalloproteinases. A marked local upregulation of both PD-1 and PD-L1, a concomitant FoxP3-positive lymphocytic infiltrate, a loss of E-cadherin, gain of epithelial–mesenchymal transition (EMT) phenotype and extreme poor differentiation at histology were also present. Upregulated microRNA in fast-growing HCCs are associated with TGF-*β* signaling, angiogenesis and inflammation. Our data show that fast HCCs are characterized not only by redundant neo-angiogenesis but also by unique features of distinctively immunosuppressed microenvironment, prominent EMT, and clear-cut activation of TGF*β*1 signaling in a general background of long-standing and permanent inflammatory state.

The molecular basis regulating tumor progression of hepatocellular carcinoma (HCC) are still unclear, although a growing evidence of data suggests that the tumor microenvironment is a key player.^1, 2, 3^ The different biochemical composition of such microenvironment derives from the underlying chronic liver disease, from the different etiologies and from the stage of the liver damage, all of this contributing to explain the high heterogeneity of HCC.^4^ The different composition and distribution of the extra-cellular matrix proteins, secreted by the activated hepatic stellate cells as consequence of the inflammatory stimuli, and how the HCC cells engage with them to negotiate their advancing across the tissue boundaries can characterize more aggressive type of cancer.^5^ The epithelial-to-mesenchymal transition is a process orchestrated by the transforming growth factor (TGF)-*β*1, stored in the ECM-enriched tissue in which HCC cells are embedded.^6^ The proteolytic remodeling of the tissue microenvironment finely modulates the dynamic cross-talk, explaining the relevance of the peritumoral tissue also as potential therapeutic target. The immunological components of the tissue microenvironment are currently considered fundamental to regulate tumoral progression, so that the programmed cell death protein 1 (PD-1)/programmed death-ligand 1 (PD-L1) immune check point is targeted by specific inhibitors in a number of different malignancies including melanoma, non-small cell lung cancer, renal cell carcinoma, head and neck cancer and bladder cancer. Most recently, clinical trials were started also in patients with advanced HCC.

All these data strongly suggest that understanding the cross-talk between the advancing tumor and the host tissue is of paramount relevance towards a precision medicine in patients with HCC. Indeed, all the most used prognostic scores, including the Barcelona Cancer Liver Center, fail to predict the clinical outcome of HCC patients in the everyday practice.^7^ Different molecular classification of HCC, derived from high-throughput studies, each of them highlighting different combination of driving genes, were recently described.^8, 9, 10, 11^ None has so far entered routine application. Recently, we have identified, a 5-genes transcriptomic signature, which accurately and significantly predicts growth speed and survival in a prospective series of HCCs at first presentation.^5^ This signature (which encounters 5 upregulated genes – angiopoietin-2 (ANGPT2), Delta-like canonical notch ligand 4 (DLL4), neuropilin and Tolloid-like 2 (NETO2), endothelial cell-specific molecule 1 (ESM1) and nuclear receptor subfamily 4 group A member 1 (NR4A1) – all associated with neo-angiogenesis) was identified through an extensive microarray study, which also evidenced a large number of other greatly dysregulated genes. Some of these associated genes suggested the possibility of a relevant imbalance of the tissue microenvironment of the HCCs identified by the transcriptomic signature.

Aim of this study was to identify the circulating and tissue components of the immune-mediated response of the microenvironment affecting the aggressiveness and the consequent clinical outcome in the HCC patients of the original cohort.^5^

## Results

To investigate the effects of the immunological tissue microenvironment components affecting the HCC clinical outcome, we performed an extensive study in the prospective cohort of 132 HCC patients, whose demographic, clinical and biochemical details were previously described^5^ and are summarized here in Supplementary Table 1. In the blood, we took into account biochemical parameters, tumoral biomarkers and cytokines; while in the tissue, we evaluated a high-throughput transcriptomics corroborated by an immunohistochemical study.

### Fast-growing HCCs have significantly higher platelet (PLT), C-reactive protein (CRP) and alpha-feto protein (AFP) levels

No significantly different values in biochemical parameters were found in the fast *versus* slow-growing HCC except in platelets count (*P*=0.023), CRP (*P*=0.036) and AFP levels (*P*=0.006). No relationship was found between higher AFP levels and etiology of liver disease (*P*=0.843), presence of portal vein thrombosis either at presentation (*P*=0.66) or during short-term follow-up (*P*=0.811), Edmondson–Steiner score (*P*=0.880), tumor-node-metastasis stage (*P*=0.783). Higher AFP levels were related with the number of nodules at presentation (*P*=0.007), lower HCC doubling time (*P*=0.007), higher TGF-*β*1 levels (*P*=0.012). At week 6 after presentation, AFP levels had significantly (*P*=0.034) increased in fast but not in slow-growing HCCs (*P*=0.726).

In the whole group, apart from TGF-*β*1, a statistically significant positive correlation was found between AFP levels and IGF-2 (*P*=0.001) and IL-8 (*P*<0.0001). In fast-growing HCCs, a positive correlation was found with adiponectin (*P*=0.020), IGF-2 (*P*<0.0001) and IL-8 (*P*=0.008) levels. In slow-growing HCCs, a negative correlation was found with angiopoietin-1 (*P*=0.024) and a positive correlation with Cyphra 21-1 (*P*=0.046).

### Fast-growing HCCs have significantly higher TGF-*β*1 and Cyphra 21-1 levels

In a large panel of 19 cytokines tested, TGF-*β*1 and Cyphra 21-1 levels were significantly higher in fast-growing HCCs (*P*<0.0001 and *P*=0.032, respectively), whereas the circulating levels of all the other cytokines were not significantly different between fast- and slow-growing HCCs (Table 1).

Overall, TGF-*β*1 levels positively correlated with VEGF (*P*<0.0001), insulin (*P*=0.023), Cyphra 21-1 (*P*=0.001) and inversely related with Visfatin (*P*=0.009). Cyphra 21-1 levels were inversely related with Visfatin only (*P*=0.001). In fast-growing HCCs, TGF-*β*1 positively correlated with Angpt-2 only (*P*=0.042). In slow-growing HCCs, TGF-*β*1 was positively related with Cyphra 21-1 (*P*<0.0001) and VEGF (*P*=0.002), and inversely related with Visfatin (*P*=0.002).

### C-type Lectin-like Receptor 2 (CLEC2) is downregulated and MMPs upregulated in fast-growing HCCs

Further analysis of hepatic Microarray data^5^ showed that several genes were differently dysregulated when the transcriptomic signature was present. In particular, in fast HCCs, we found a striking downregulation of CLEC2 and upregulation of several metalloproteinases (MMPs)(Supplementary Table 2). CLEC2 is a non-classical C-type lectin-like receptor, encoded by the *CLEC1B* gene. It is expressed in myeloid cells (including monocytes, dendritic cells, NK cells and granulocytes), liver sinusoidal endothelial and Kupffer cells but it has recently been found also in epithelial cells (and stomach). CLEC2 has a known exogenous (rhodocytin) and an endogenous ligand (podoplanin) but other ligands are likely to exist. It has been shown that CLEC2 is involved in lymphatic/blood vessel separation, tumor cell-induced platelet aggregation, immune response and epithelial–mesenchymal transition (EMT).

Hepatic CLEC2 expression was strongly downregulated in fast compared to slow-growing HCCs (*P*<0.0001). Other genes, involved in AKT signaling, were notably dysregulated. GSK3A (*P*=0.0069), and eIF4E (*P*=0.02) were significantly upregulated while PTEN (*P*=0.01) and FoxO (*P*=0.0019) were significantly downregulated in fast *versus* slow HCCs.

In contrast, expression of MMPs was upregulated in fast respect to slow-growing HCCs, in particular MMP1 (*P*<0.0001), MMP10 (*P*<0.0001) and MMP12 (*P*<0.0001). A strong inverse relationship was present between hepatic CLEC2 RNA expression and the different MMP RNAs (MMP-1: *P*=0.002, MMP-10: *P*=0.045, MMP-12: *P*=0.016, respectively).

**Figure 1:**
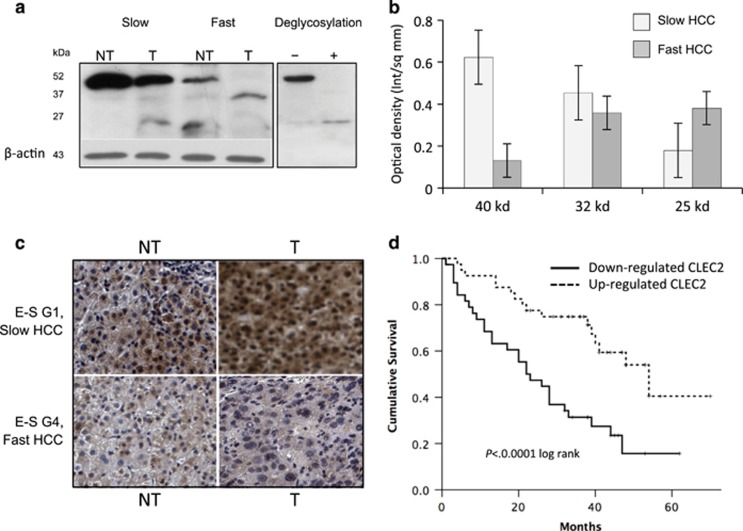
(**a**) Representative WB analysis of CLEC2 in fast and slow and HCCs. Low molecular weight forms of the protein (∼32 and ∼ 25 kD) were mostly present in fast HCC, while full-length forms (∼40 kD) were more often present in slow HCCs. These lower bands are consistent with deglicosylated forms. (**b**) Distribution of different CLEC2 forms in fast and slow HCCs. Columns represent mean of optical density of different isoforms (40, 32 and 25 kD), normalized to *β*-actin, in slow and fast HCC subgroups (error bars: S.D.). On the whole, 20 patients (14 with slow tumors and six with fast tumors) were evaluated. (**c**) Immunohistochemical staining of CLEC2 in the liver shows marked downregulation of CLEC2 in fast, poorly differentiated HCCs, while slow HCCs displays significantly higher levels of expression. (**d**) Survival analysis by Kaplan–Meier shows significantly lower survival in HCC patients with downregulated CLEC2. E–S: Edmondson–Steiner

Interestingly, CLEC2 downregulation at RNA level was significantly related with presence, at western blot, of low molecular weight forms of the protein (∼32  and 25 kD) and marked decrease or disappearance of the full-size protein (∼40 kD) (Figures 1a and b) with the presence, in a few cases, of the 25 kD isoform only. These monomeric low molecular weight CLEC2 forms, corresponding to forms lacking one or two of the three glycosylation sites (as indicated by results of deglycosylation) (Figure 1a), were mostly found in tumor tissue of poorly differentiated HCCs, especially, but not exclusively, when belonging to the fast phenotype (Figure 1c). In non-tumor cirrhotic tissue (especially in slow-growing HCCs), CLEC2 isoforms were normally glycosylated.

At histochemical level, lower CLEC2 expression significantly related with poorly differentiated histology (mean optical density G1-3 *versus* G4 Edmondson score: 0.24±0.12 *versus* 0.15±0.07, *P*=0.033, Mann–Whitney). CLEC2 RNA levels were also inversely related with circulating TGF-*β*1 levels, the highest levels of the latter being found in HCCs in which CLEC2 was strikingly downregulated (*P*<0.0001).

### CLEC2 downregulation and MMPs upregulation correlate with clinical outcome

Down- or upregulated genes affected prognosis and survival. Upregulation of MMP-1, MMP-10 and MMP-12 was associated with significantly decreased survival (Supplementary Figure 1). CLEC2 downregulation was significantly related with survival, median survival of HCCs with downregulated CLEC2 being 22 months *versus* 54 months (*P*<0.0001)(Figure 1d). For patients with downregulated CLEC2, the concomitant presence of the transcriptomic signature was associated with a further decrease of median survival. Fast HCCs with CLEC2 lower than median levels had a median survival of 13 months *versus* 27 months (upregulated CLEC2 and aggressive signature), 44 months (downregulated CLEC2 without aggressive signature) and 48 months (upregulated CLEC2 and slow HCC) (*P*<0.0001) (Supplementary Figure 2A). Very low survival was also observed in patients who displayed downregulated CLEC2 and poorly differentiated histology. Patients with downregulated CLEC2 and Edmondson–Steiner (E–S) G4 had a median survival of 7 months *versus* 26 (CLEC2 downregulated/E–S G1-3), 26 (CLEC2 up/E-D G4) and 52 months (CLEC2 up/E–S G1-3), respectively (*P*<0.0001 for all) (Supplementary Figure 2B). Further lower survival was observed when downregulated CLEC2, E–S G4 and transcriptomic signature were present together (median survival 6 months) (*P*<0.0001) (Supplementary Figure 2C).

### CLEC2 correlates with low E-cadherin expression in fast-growing HCCs

**Figure 2:**
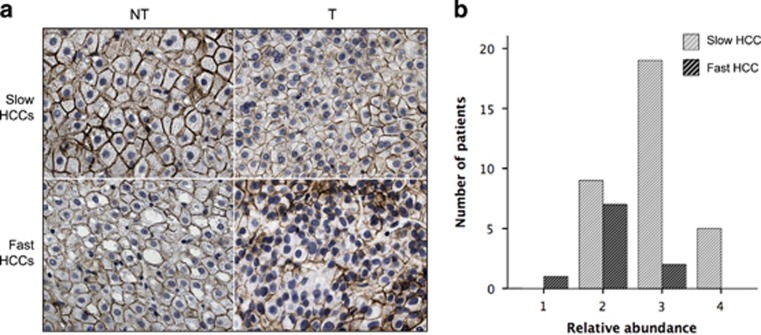
Immunohistochemical analysis of E-cadherin in slow and fast HCCs. (**a**) E-cadherin membrane localization was preserved in non-tumoral cirrhotic tissue and to a lesser extent in tumoral tissue in slow HCC. (**b**) The relative abundance of E-cadherin-positive cells was graded from 0 to 4 by counting with ImageJ software (http://rsbweb.nih.gov/) at least 100 tumor cells in areas of heterogeneous E-cadherin expression (0=less than 5% of positive cells; 1=5–25% 2=26–50% 3=51–75% and 4=76–100%). Forty-three patients were studied (33 with slow HCC, 10 with fast HCC) and the bars represent the number of patients with the different score. Data reported are from tumor tissue. In fast HCC, E-cadherin membrane expression was reduced in non-tumoral tissue and almost absent in tumoral tissue (*P*=0.014)

Expression of E-cadherin was not significantly different in non-tumoral hepatic tissue between patients with fast and slow HCC (Figure 2a). E-cadherin positive cells were instead significantly decreased in tumoral tissue both as intensity and number of positive cells: the loss of E-cadherin membrane expression was significantly higher in patients with fast HCCs than in slow HCCs (*P*=0.014) (Figures 2a and b). Loss of E-cadherin expression was significantly related with CLEC-2 downregulation in the tumor, both at transcriptional (*P*=0.005) and post- transcriptional level (*P*=0.010).

At RNA level, CDH1 gene was significantly downregulated in tumoral tissue from fast HCCs *versus* slow ones (*P*=0.002). In non-tumoral tissue the difference did not reach significance (*P*=0.096).

### MicroRNA in fast-growing HCCs are associated with TGF-*β* signaling, angiogenesis and inflammation

To investigate how gene expression is regulated, we evaluated a group of 82 microRNA, selected for being associated with regulation of angiogenesis, cell proliferation, cell signaling, immune control and autophagy (Supplementary Table 3).

**Figure 3:**
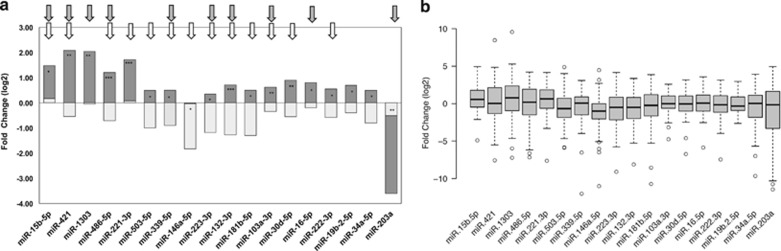
MicroRNA expression in fast and slow HCCs. (**a**) All microRNAs but 146a-5p and 203a were significantly overexpressed in fast HCCs (level of significance: *P<0.01; **P<0.001; ***P<0.0001). Arrows indicated the relationship of each miRNA with survival: white arrows: significantly associated with survival at 12 months gray arrows: significantly associated with survival at the end of follow-up. (**b**) MicroRNAs expression in all HCCs *versus* surrounding non-tumoral cirrhotic tissue, non stratified in fast and slow HCCs

Eighteen microRNAs were differentially expressed in tumor tissue of fast HCCs in comparison with slow ones (Figure 3). All these 18 microRNAs but two (146a-5p and 203a) were upregulated in fast HCCs; in particular, 203a was strongly downregulated. In slow HCCs, all these microRNAs were either downregulated or not differentially expressed from cirrhotic tissue (Figure 3b).

Five microRNA were markedly upregulated in fast HCC while they were downregulated in slow HCCs: mir-15b-5p, mir-421, mir-1303, mir-221-3p and mir-486-5p. These microRNAs are involved in TGF-*β*/BMP-signaling pathway, angiogenesis, autophagy and inflammation.

A significant relationship between 16 microRNAs dysregulation and survival was found: downregulation of these microRNAs in slow HCCs was associated with significantly better survival, while their upregulation was associated with significantly worse short- and long-term survival (Supplementary Figure 3). The only exception to that was mir-203a, whose downregulation in fast HCCs was associated with significantly worse survival. Lower levels of mir-203a were also significantly associated with loss of E-cadherin membrane expression in the tumor (*P*=0.028).

**Figure 4:**
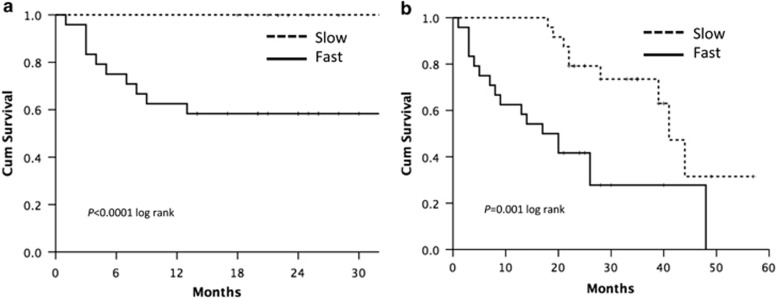
Survival analysis by Kaplan–Meier: the five microRNA signature tested at presentation was able to predict survival both at 12 months (**a**) and at the end of follow-up (**b**)

A signature composed of the five microRNAs most significantly associated with survival was able to predict survival both at 12 months and at end of observation (Figure 4).

### PD-1/PD-L1 is more expressed in fast-growing HCCs

To investigate the immune cells infiltrating tissue microenvironment, we evaluated in paired tumor and non-tumor hepatic biopsies the expression of PD-1 and PD-L1 and FoxP3.

**Figure 5:**
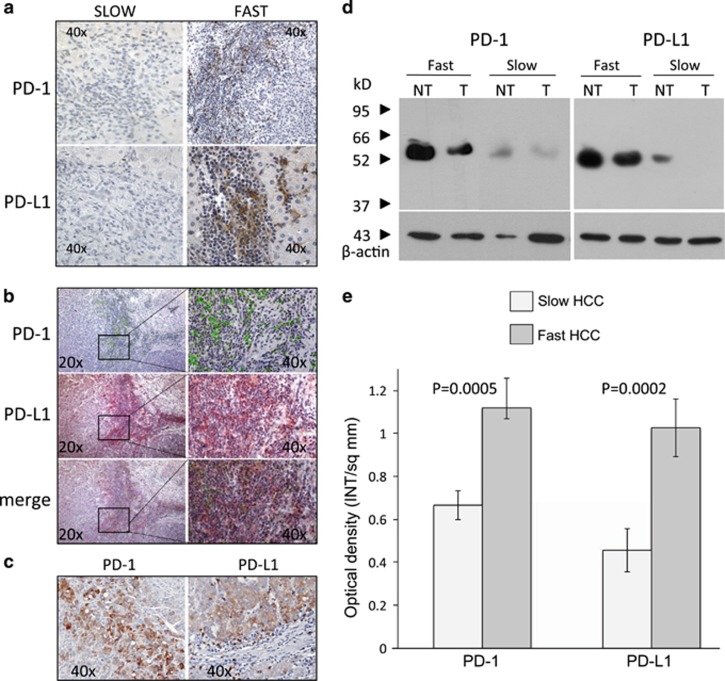
(**a**) Immunohistochemical demonstration of PD-1 and PD-L1 in slow and fast HCCs. Fast HCCs display strong positivity, localized in the lymphocytic infiltrate. (**b**) PD-1- and PD-L1-positive cells co-localize in the areas of stronger lymphocytic infiltrate. (**c**) In fast HCC, both PD-1 and PD-L1 can be demonstrated in hepatocytes. (**d**) Western blot analysis confirms the higher level of positivity of fast HCCs in comparison with slow HCCs, which are weakly positive or negative in the tumor. (**e**) Semi-quantification of PD-1 and PD-L1 in tumor tissue of fast and slow HCCs. Columns represent mean of optical density, normalized to *β*-actin, of 20 patients (14 with slow tumors and six with fast tumors) (error bars: S.D.). PD-1 and PD-L1 levels were significantly different between slow and fast HCCs (*P*=0.0005 and *P*=0.0002, respectively)

PD-1 was not only expressed in the lymphocytes infiltrating the tumor but also on neoplastic hepatocytes (more often with a membranous expression but, to a lesser extent, cytoplasmic) (Figures 5a and c). PD-1 positive lymphocytes were almost absent in the lobular region, being mostly localized in fibrous septa and in the portal tract. In patients with slow HCC, PD-1 expression in tumor tissue was very weak to negative while it was strongly positive in most fast HCCs (*P*=0.001) (Figures 5a and b). Expression in NT tissue was much weaker, although patients with fast HCC displayed relevant PD-1 expression also in non-tumoral tissue (Figures 5a and b).

PD-L1 was expressed both in tumoral hepatocytes, in infiltrating lymphocytes cells and in sparse macrophages. Positive cells were also present in the lobular region. As for PD-1, much higher PD-L1 expression was found in aggressive HCCs in comparison with slow ones, in which it was weak to negative (*P*=0.013) (Figures 5a and e). PD-1 co-localized with PD-L1, especially in areas of major lymphocyte infiltration (Figure 5b) and with FoxP3. A positive correlation was found between degree of infiltrating lymphocytes and levels of PD-1 and PD-L1 positivity (*P*=0.001 and *P*=0.029, respectively). Stratifying by biological aggressiveness, the correlation between lymphocytes infiltration and PD-1 positivity hold true only for aggressive HCC (fast HCC: *P*=0.048; slow: *P*=0.275).

PD-L1 mRNA expression was in agreement with protein expression: CD274 RNA was significantly more expressed in high-risk signature HCCs (*P*=0.004) in tumoral but not in non-tumoral tissue (*P*=0.818). Western blot analysis confirmed the high level of expression of both PD-1 and PD-L1 in patients with fast HCC, while in slow HCC they were barely expressed (Figure 5d).

## Discussion

The heterogeneity of HCC represents a major obstacle for understanding the mechanisms underlying its onset and its progression.^4^ Different studies report opposite or discordant alterations of the same genes or pathways. Possible explanations are the retrospective nature of such studies^12^ and surgical resection as a source of tissues specimens, with an obvious bias of selection among the whole population of patients. Finally all the previous studies are informative on the situation at the moment of tissue collection, that is, at resection, thus representing a snapshot of the disease at heterogeneous time points of the clinical history. In contrast, herein we conducted the analysis of the immunological cellular components of the microenvironment on tissues obtained by needle biopsy at HCC presentation, in patients subsequently followed in a longitudinal prospective study. In this recent study,^5^ we chose growth speed as indicator because previous studies from our group^13^ and others^14, 15^ showed that growth speed strongly relates with progression and prognosis of HCC. Patients with a tumor doubling time lower than 53 days carried the neo-angiogenic transcriptomic signature, which strongly related with rapid progression of tumor, risk of recurrence after therapy and with extremely low median survival.^5^ In the present study, we focused on the characterization of the microenvironment of HCC in relation with the presence of the neo-angiogenic transcriptomic signature. We have found that the biology of fast, aggressive HCCs is profoundly different from that of the slow subtype. Fast HCCs display a markedly immunosuppressed microenvironment (as shown by the local upregulation of both PD-1 and PD-L1) in a background of higher systemic inflammation, with a distinct switch toward EMT and extreme poor differentiation at histology. In aggressive HCCs, a set of MiRNA known to be downregulated or non differentially expressed from non-tumoral cirrhotic tissue in unstratified HCCs was found to be paradoxically upregulated in fast HCCs, contributing to the overall loss of control of immune regulation, angiogenesis and proliferation.

One of the main findings pointing toward increased local immunosuppression is the finding of marked PD-1/PD-L1 overexpression. Long-standing inflammation, which is the common background for both viral and non-viral associated chronic liver disease, favors cytotoxic T-cell exhaustion and taking over of T-regs (as shown by high FoxP3 expression). We do not have means of evaluating whether the inflammatory activity throughout the course of disease had been greater in aggressive HCCs. However, systemic inflammation, as evaluated by increased CRP and platelets levels,^16, 17^ was significantly higher in fast HCCs compared with slow ones. Only few studies have examined PD-1/PD-L1 in liver cancer. Recently, a study performed in resected HCC specimens showed that PD-L1 expression in either neoplastic or intratumoral inflammatory cells related with macrovascular and microvascular invasion and poor differentiation.^18^ PD-1 expression was found only in infiltrating lymphocytes. The Authors or in alternative, they concluded that these features related with HCC characterized by higher AFP levels and lymphoepithelioma-like histological subtype. These results are in agreement with our findings. In our prospective series of HCC studied at presentation, we demonstrated that the most aggressive HCCs (defined by the transcriptomic signature) have relevant expression of PD-1/PD-L1 while slow HCCs do not. As not more than 20% of HCCs carry the transcriptomic signature, this means that PD1/PD-L1 would be definitely positive in about 1/5 of cases only. This has implications for therapeutic strategies with PD-1/PD-L1 inhibitors: although there are indications that check point inhibitors have some activity independently from PD-1/PD-L1 status, strongly positive cases are reported to have higher response.^19^ We also found intense PD-1 expression not only in infiltrating lymphocytes but also on tumoral hepatocytes. This is a novel finding in HCC. There are reports, however, of the presence of PD-1 on tumor cells in other tumors with a relevant inflammatory background like melanoma.^20^ Inflammation has been shown to be a key driver also for the induction of PD-L1.^21^ What is clear from our data is the strict relationship between entity of lymphocytic infiltrate, PD1/PD-L1 positivity and aggressive HCC phenotype. This has clear therapeutic implications: these patients have an extremely severe prognosis, especially if they have also poorly differentiated histology, with a median survival as low as 6 months. Checkpoints inhibitors could represent a substantial chance of cure for these patients.

Another striking finding in this cohort of HCCs was the marked downregulation of CLEC2. CLEC2 is a non-classical C-type lectin-like receptor, which is expressed on platelet and hematopoietic cells.^22, 23^ CLEC2 localization in epithelial cells has been rarely reported.^24^ Interestingly, the recent study by Wang *et al.*^25^ showed that CLEC2 is present in normal gastric mucosa but is downregulated in gastric tumors. CLEC2 downregulation relates with depth of tumor invasion, metastasis to lymph node, and 5-year survival of patients. They concluded that CLEC2 inhibits Akt signaling by blocking expression of phosphoinositide 3-Kinase and may act as a suppressor of tumor transformation and metastasis in gastric cancer. Our data in HCC are consistent with those by Wang *et al.*^25^ in gastric cancer. We showed that CLEC2 is abundantly expressed in non-tumoral liver tissue in fully glycosylated form, while its expression is markedly decreased or absent in HCC, and present in bi- or mono-glycosylated form. Furthermore, CLEC-2 was downregulated in liver cancer at both transcriptional and post-translational levels; this significantly related with upregulation of genes related with Akt signaling like GSK3 and eIF4E, with PTEN downregulation, with poorly differentiated histology (i.e., G4 in the Edmondson–Steiner classification), aggressive HCC phenotype bearing the transcriptomic signature and evidence of EMT. These patients had a median survival of 6 months.

The pattern of microRNA expression was also very distinctive. Only 17 out of 82 microRNAs tested were differentially expressed in fast in comparison with slow HCCs. All microRNAs identified but two (mir-146a-5p and miR-203a) were overexpressed in aggressive HCCs and downregulated in slow HCCs. The top five overexpressed microRNAs (mir-15b-5p, mir-421, mir-1303, mir-221-3p and mir-486-5p) were very informative of the activated pathways in these HCCs. Two of them (mir-15b-5p and mir-421) are involved in TGF*β*1 pathway and in inflammatory signaling;^26, 27^ mir-15b-5p is also involved in G1-S transition.^28^ Mir-1303 modulates proliferation, migration and cell invasion and differentially controls Glypican-3 expression in HCC cells.^29^ Mir-221-3p is involved in cell cycle progression and has a oncogenic role in gastric carcinoma.^30, 31^ Mir-486-5p has a role in angiogenesis, lymphangiogenesis, and cell proliferation.^32, 33^ For some of these microRNAs, discordant indications on their role in HCC exist. Increased mir-15b-5p was negatively correlated with HCC recurrence.^34^ Yang *et al.*^35^ suggested that mir-15b-5p acted as a tumor suppressor gene in HCC inducing endoplasmic reticulum stress, apoptosis, and growth inhibition by targeting and suppressing Rab1A. However, Liu *et al.*^27^ found elevated mir-15b-5p in HCC tissue and in the serum, which decreased after curative resection, thus suggesting a causal role for mir-15b-5p in HCC progression. Mir-486-5p was previously reported as downregulated in several cancers, among which HCC; the low mir-486-5p expression in HCC tissue was associated with high recurrence rate and poor disease-free survival after tumor resection.^36^ Experimental overexpression of mir-486-5p markedly suppressed HCC cell proliferation and inhibited HCC growth *in vivo*. These findings are in contrast with our data. We found a significantly worse survival in patients, also carrier of the transcriptomic signature, with upregulated mir-15b-5p or mir-486-5p. A partial explanation can be the different composition of the patients’ cohorts. Studies with discordant results^34, 35, 36^ investigated HCC samples derived from resection, representative of the small percentage of slow-growing HCCs liable to this type of therapy but having a different biological behavior than the fast ones. Most important, however, is the fact that fast HCCs are similar to other aggressive inflammatory cancers, which show microRNA dysregulation concordant with our results. In these cancers, like sebaceous carcinoma^37^ or glioma^38^ a strong relationship between overexpressed mir-486-5p and worse outcome was found. In this context, mir-486-5p represses several regulators of nuclear factor (NF)-KB, thus favoring persisting inflammation and further cytotoxic T-cell exhaustion. To further underline the relevance of the inflammatory background in determining the pattern of microRNA dysregulation, mir-486-5p together with mir-421, mir-1303, mir-221-3p (all notably upregulated in fast HCCs) are comprised in a 5-gene microRNA signature, predictive of breast tumor aggressiveness, recently described in inflammatory breast cancer (IBC).^39^ This signature, which accurately predicts IBC phenotype with an overall accuracy of 89%, identifies a type of breast cancer that shares many features with the fast HCCs of our cohort, that is, intense angiogenesis, high proliferation and metastatic ability. IBCs are also characterized by transcriptomic signatures strongly upregulated for NF-KB-dependent genes and downregulated for estrogen-dependent genes, a feature also present in our fast HCCs (data not shown). On the whole, these characteristics underline the extreme importance of the inflammatory background of liver disease, with many of the microRNAs involved with inflammation having a role also in angiogenesis. Most interestingly, the only significantly downregulated microRNA in fast HCCs was mir-203a, which has been reported to act as an anti-oncogene to suppress HCC tumorigenesis and to be a marker of poor prognosis of HCC when downregulated.^40, 41^ Loss of miR-203a results in activation of EMT in normal hepatocytes and HCC tumor cells and is associated with prominent downregulation of E-cadherin expression, as we showed in fast HCCs.^40^

**Figure 6:**
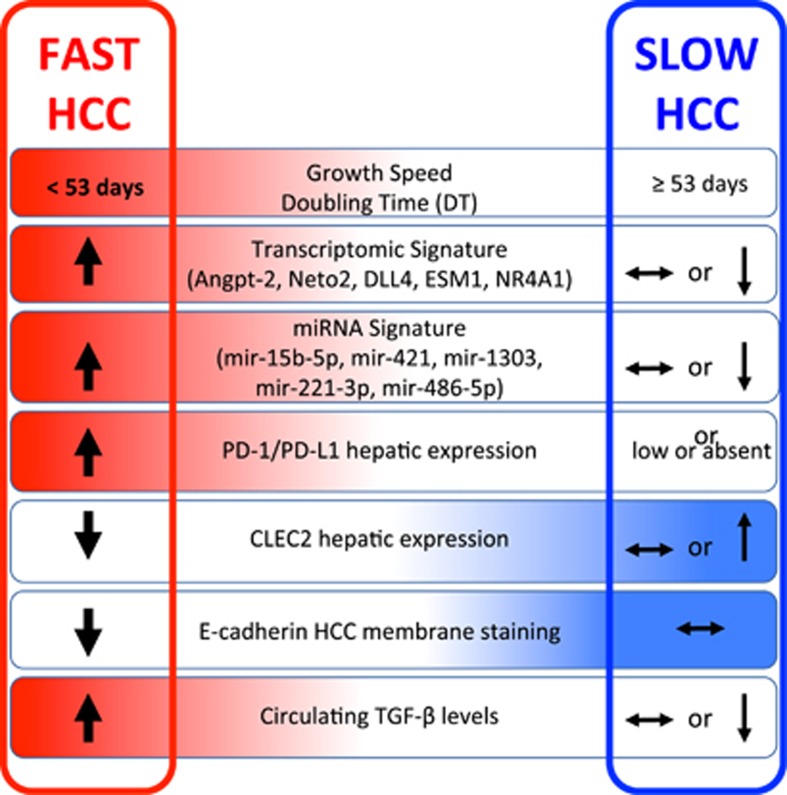
A graphic abstract showing the major findings of this study. The distinctive features of fast and slow HCCs are depicted

In conclusion, we have characterized the circulating and tissue components of the microenvironment of fast HCCs. Apart from redundant neo-angiogenesis,^5^ fast HCCs bear very unique features of distinctively immunosuppressed microenvironment, prominent EMT, and noticeable activation of TGF*β*1 signaling in a general background of long-standing and permanent inflammatory state (Figure 6). This suggests that every effort should be made throughout the long natural history of patients with liver disease to switch off the inflammatory process, whatever the cause, as early as possible.

## Materials and methods

The patients investigated in this study were those previously reported in a prospective genome-wide transcriptomic study, in which a 5-gene signature was identified which accurately defined HCC growth rate and patients’ survival characteristics.^5^ Growth rate was studied by a specifically set up imaging protocol and internally validated in an independent prospective cohort.^5^ Demographic, clinical and biochemical details of the training and validation cohorts are reported in Supplementary Table 1. Enrollment in the study started in 2008; median follow-up was 36 months.

### Determination of serum cytokines

Interleukin (IL)-1 alpha, IL-1beta,, IL-10, tumor necrosis factor (TNF)-alpha (Aushon Biosystems, Billerica, MA, USA), Adiponectin (DRG International, Springfield, NJ, USA; NJ, USA), Visfatin (Phoenix Pharmaceutical, Inc., Burlingame, CA, USA)., Insulin-like growth factor (IGF)-2 (Boster Biological Technology, Pleasanton, CA, USA), and Hepatocyte growth factor (HGF) (Aushon Biosystems, Inc., Billerica, MA, USA), were measured in duplicate, according to the manufacturer’s instructions. Serum CYFRA21-1 was tested with the human cytokeratin fragment antigen 21-1 (CYFRA21-1) ELISA kit (Cusabio Biotech Co., Ltd, Wuhan, P.R. China) according to the manufacturer’s instructions. Serum IL-6, TNF-alpha, IL-8, VEGF, and TGF-*β*1 levels were determined with the quantikine/high-sensitivity enzyme-linked immunosorbent assay kit (R&D Systems, Minneapolis, MN, USA), according to the manufacturer’s instructions.

Absorbances were measured at 450 and 490 nm with an automatic microplate reader (Multiskan EX; Thermo Fisher Scientific, Inc, Waltham, MA, USA), with background subtraction at 570 and 650 nm, respectively.

### Immunohistochemistry

Immunostaining was performed on 5-*μ*m sections of formalin-fixed and paraffin-embedded tissues of the original biopsies obtained at presentation, also evaluated for transcriptomic and microRNA analysis. Sections were incubated for 30 min at 98 °C with 10 mM citrate buffer pH 6.0 or EDTA buffer pH 8, depending on antibody used, for antigen retrieval, treated with 3% hydrogen peroxide for 10 min and blocked using a blocking solution reagent for 1 h at room temperature. Sections were then incubated with either (a) rabbit monoclonal antibody for PD-L1 (E1L3N) (Cell Signaling Technology, Inc., Danvers, MA, USA) at working dilution of 1:80/rabbit monoclonal antibody for PD-1 (D4W2J) diluted 1:100 at 4 °C overnight; (b) mouse monoclonal antibody for FoxP3 (1:50, clone 236A/E7; eBioscience, San Diego, CA, USA); (c) mouse polyclonal antibody for C-type lectin-like receptor 2 (CLEC-2 /CLEC1B) (working dilution 1:80) (Ventana Medical Systems, Tucson, AZ, USA); or (d) with pre-diluted (ready-to-use) mouse monoclonal antibody against E-cadherin (Ventana Medical Systems, Tucson, AZ, USA) at 37 °C for 24–38 min. Sections were then incubated with detection kit reagents (Ultra view universal HRP multimer and Diaminobenzidine (DAB) Chromogen, Ventana Medical Systems, Inc., Tucson, AZ, USA) following the manufacturer’s instructions. The sections were then counterstained with hematoxylin,dehydrated and permanently mounted for microscopic examination. Images of stained liver tissue were processed with ImageJ software (http://rsbweb.nih.gov/) to obtain the medium intensity value of DAB signal. Three fields were chosen at random under 200-fold magnification; the number of stained cells for each intensity level was counted. Cases were scored as weak, intermediate, and strong brown staining. The expression score was calculated according to the formula: =0 × % of non-stained tumor cells + 1 × % of weakly stained tumor cells + 2 × % of moderately stained tumor cells + 3 × % of strongly stained tumor cells. An expression score between 0 and 30 was obtained where 30 was equal to 100% of tumor cells strongly stained.^42, 43^ Two pathologists independently performed the semi-quantitative estimation of immunoreactive cells, based on staining intensity and relative cellular abundance.

### MicroRNA

#### RNA Extraction and real-time PCR analysis

The total RNA used for this study was a portion of the RNA used for the published transcriptomic analysis.^5^

Mature microRNAs extracted from liver tumor and non-tumor tissue were reverse transcribed and amplified using the miRCURY LNATM Universal RT microRNA PCR system (EXIQON, Inc., Woburn, MA, USA). qRT-PCR was performed in a LightCycler 480 instrument (Roche, Mannheim, Germany) using miRCURY LNATM SYBR Green master mix (EXIQON) and Custom microRNA LNATMPCR primer set (EXIQON) in 10 *μ*l reaction, at volume of 10 ng complementary DNA (cDNA)/RNA concentration. The reactions were incubated in a 96-well plate at 95 °C for 10 min, 45 cycles of 95 °C for 10 s, 60 °C for 1 min. Each sample was analyzed in duplicate. The level of microRNA expression was measured using Ct (threshold cycle). The 2-ΔΔCt method for relative quantification of gene expression was used to determine miRNA expression levels. The ΔCt was calculated by subtracting the Ct of U6 and RNAU5G from the Ct of the microRNA of interest. Using this method, we obtained the fold changes of gene expression normalized to two internal control genes and relative to the corresponding cirrhotic non-tumor liver tissue.

### Western blot analysis

Liver tissue was completely dissociated with Gentlemax Dissociator (Miltenyi Biotec GmbH, Bergisch Gladbach, Germany), according to the manufacturers' instructions, in ice-cold RIPA buffer, supplemented with protease inhibitors (Complete; Roche Applied Science, Mannheim, Germany) and phosphatase inhibitors (PhosSTOP; Roche Applied Science). Protein concentration was determined by BCA method (Quantum micro protein kit; Euroclone Spa, Pero, MI, Italy). 20 *μ*g of protein extracts were subjected to reducing 10% sodium-dodecyl sulfate-polyacrylamide gel electrophoresis and transferred to a PVDF membrane (GE Healthcare, Amersham, UK), which was probed with CLEC2/CLEC1b-specific Antibody (MAB1718; R&D System, Minneapolis, MN, USA), PD-1 (D4W2J) XP Rabbit mAb (#86163, Cell Signaling Technology, Inc., Danvers, MA, USA) and PD-L1 (E1L3N) XP Rabbit mAb (#13684, Cell Signaling Technology, Inc.) overnight at 4 °C, after being blocked with a 5% solution of nonfat dry milk dissolved in blotting buffer (25 mM Tris, 0.15 M NaCl, 0.1% Tween 20, pH 7.4) or T. Membranes were subsequently washed and exposed for 1 h at room temperature to a secondary mouse IgG horseradish peroxidase-conjugated antibody (HAF018; R&D System) and to a secondary rabbit IgG horseradish peroxidase-conjugated antibody (SC2004; Santa Cruz Biotechnology, Inc., Dallas, TX, USA) and detected by chemiluminescence (LiteAblot Plus; Euroclone Spa, Pero, MI, Italy) followed by autoradiography and densitometry analysis by Quantity One 1-D Analysis Software (BioRad, Hercules, CA, USA).

#### Enzymatic deglycosylation

50 *μ*g of total protein extract was denatured in 0.1% SDS, 0.1 M 2-Mercaptoethanol buffer, for 5 min at 95 °C. The samples were incubated for 4 h at 37 °C with N-glycosidase F (Roche Applied Science) in deglycosylation buffer (400 mM Sodium phosphate mono/dibasic, pH 7.4) with the addition of protease inhibitor (Complete; Roche Applied Science) and 0.8% v/v Nonidet P40 (Roche applied Science) as described by Viapiano Lab (Boston, MA, USA), 2007. Bovine fetuin (Sigma Aldrich, Inc., St. Louis, MO, USA) was used as a control glycoprotein. Enzyme reactions were subsequently analyzed by SDS-PAGE and western Blot analysis.

### Statistical analysis

Dichotomous and continuous variables were compared using Fisher’s exact test and the nonparametric Mann–Whitney *U-*test, respectively.

The Kaplan–Meier method was used to estimate the cumulative probability of overall survival. Patients were censored at the time of LT, death or the last available follow-up. Differences in observed probability were assessed using the log-rank test.

The PASW Statistics 20 program (IBM Corp., Armonk, NY, USA) was used for statistical analyses.

All the authors had access to the study data and reviewed and approved the final manuscript.

## Publisher’s Note

Springer Nature remains neutral with regard to jurisdictional claims in published maps and institutional affiliations.

## Figures and Tables

**Table 1:** Circulating levels of all the cytokines tested, stratified by slow and fast HCC (S.D.)

	**Slow HCCs (Mean±S.D.)**	**Fast HCCs (Mean±S.D.)**	***P***
Angpt1 (ng/ml)	24.389±12.796	25.280±15.482	0.80
Angpt2 (ng/ml)	6.470±4.052	6.471±4.389	0.94
VEGF	288±273	395±311	0.10
Insulin	14.6±7.4	19.5±13.8	0.47
Adiponectin	13.6±9.2	14.1±7.4	0.47
Leptin	11.1±12.4	13.2±9.8	0.75
Visfatina	13.6±8.4	12.1±5.7	0.58
IGF2	8.5±22.0	6.9±17.7	0.20
TGF*β*1 (ng/ml)	5.718±3.563	13.111±8.086	**0.00**
CYFRA21-1 (ng/ml)	6.0±2.9	7.6±3.2	**0.03**
HGF	1.775±1.046	1.639±755	0.84
IL_1a (pg/ml)	7.1±16.0	8.2±17.3	0.61
IL_1b (pg/ml)	2.1±4.9	4.9±15.66	0.75
IL_6 (pg/ml)	47.1±64.8	84.2±184.9	0.96
IL_8 (pg/ml)	49.6±59.3	79.0±91.0	0.13
IL_10 (pg/ml)	7.1±14.8	3.5±3.2	0.76
TNFα (pg/ml)	4.8±12.0	4.8±9.0	0.21

S.D., standard deviation. Bold values indicate significant values.
