# Supplementary material for: Microenvironment inflammatory infiltrate drives growth speed and outcome of hepatocellular carcinoma: a prospective clinical study
Source: Cell Death Dis. 2017 Aug 24;8(8):e3017–. doi: 10.1038/cddis.2017.395 (PMC5596578; doi:10.1038/cddis.2017.395)

Survival according to median levels of MMP1, MMP10 and MMP12. These MMPs were over-expressed in fast HCC. Worse survival was observed in patients with higher median levels

MMP1

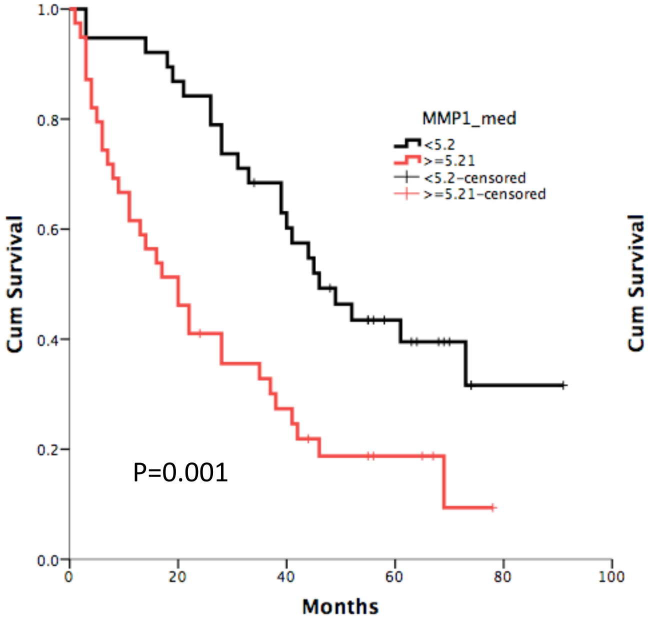

MMP10

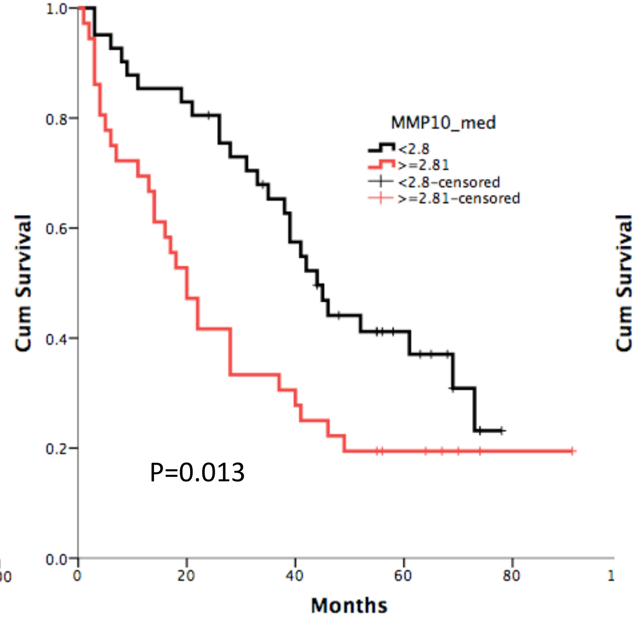

MMP12

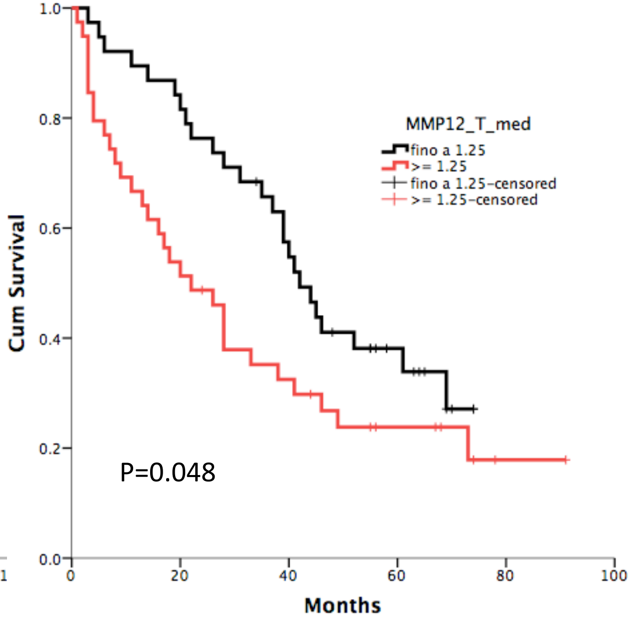

Supplement: Supplementary Figure 1 [file cddis2017395x1.pdf]
