# Supplementary material for: Microenvironment inflammatory infiltrate drives growth speed and outcome of hepatocellular carcinoma: a prospective clinical study
Source: Cell Death Dis. 2017 Aug 24;8(8):e3017–. doi: 10.1038/cddis.2017.395 (PMC5596578; doi:10.1038/cddis.2017.395)
Supplement: Supplementary Figure 2 [file cddis2017395x2.pdf]

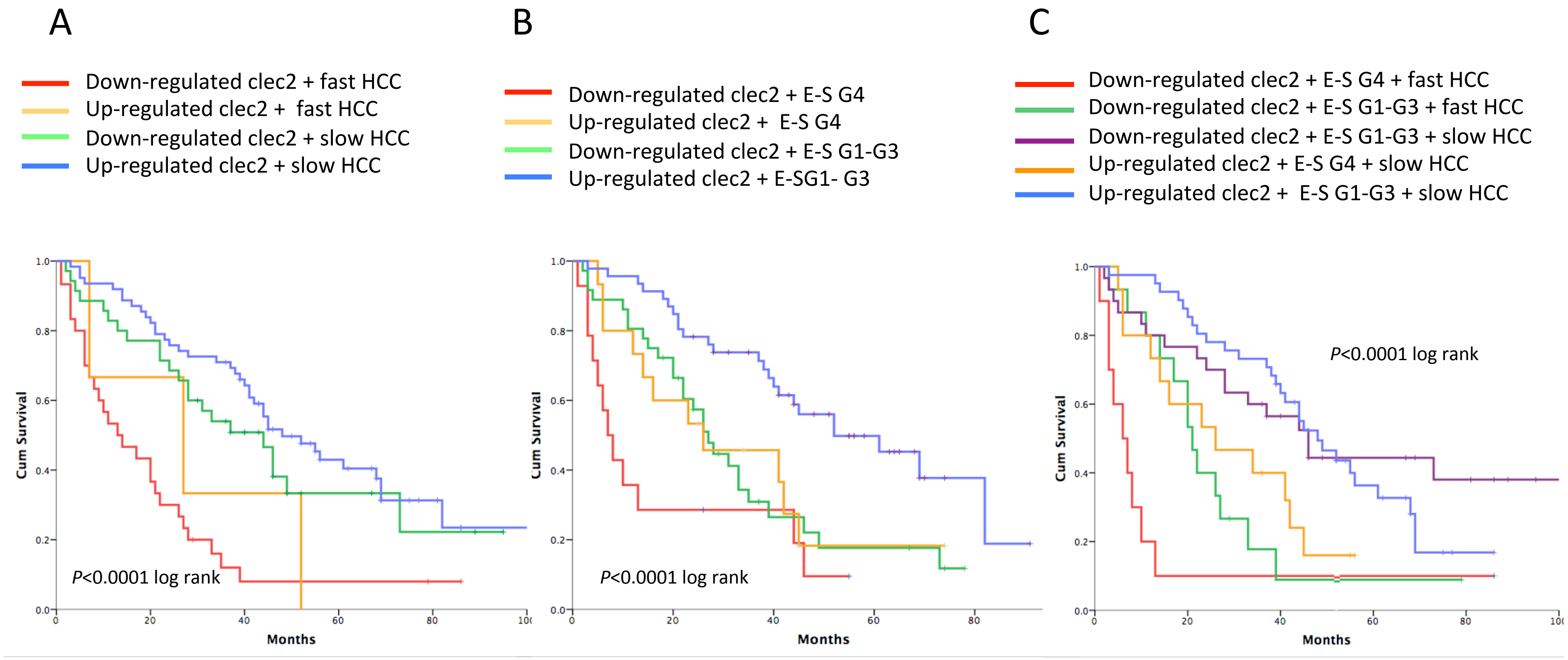
A
Down-regulated clec2 + fast HCC
Up-regulated clec2 + fast HCC
Down-regulated clec2 + slow HCC
Up-regulated clec2 + slow HCC
Cum Survival
Months
P<0.0001 log rank
B
Down-regulated clec2 + E-S G4
Up-regulated clec2 + E-S G4
Down-regulated clec2 + E-S G1-G3
Up-regulated clec2 + E-SG1- G3
Cum Survival
Months
P<0.0001 log rank
C
Down-regulated clec2 + E-S G4 + fast HCC
Down-regulated clec2 + E-S G1-G3 + fast HCC
Down-regulated clec2 + E-S G1-G3 + slow HCC
Up-regulated clec2 + E-S G4 + slow HCC
Up-regulated clec2 + E-S G1-G3 + slow HCC
Cum Survival
Months
P<0.0001 log rank
