# Supplementary material for: Microenvironment inflammatory infiltrate drives growth speed and outcome of hepatocellular carcinoma: a prospective clinical study
Source: Cell Death Dis. 2017 Aug 24;8(8):e3017–. doi: 10.1038/cddis.2017.395 (PMC5596578; doi:10.1038/cddis.2017.395)
Supplement: Supplementary Figure 3 [file cddis2017395x3.pdf]

Survival according to median levels of MicroRNA, identified as differentially expressed in fast and slow HCCs (A: prediction of long term survival ; B : prediction of short term survival). Worse survival was observed in patients with higher median levels of all Mirna but Mir-203a

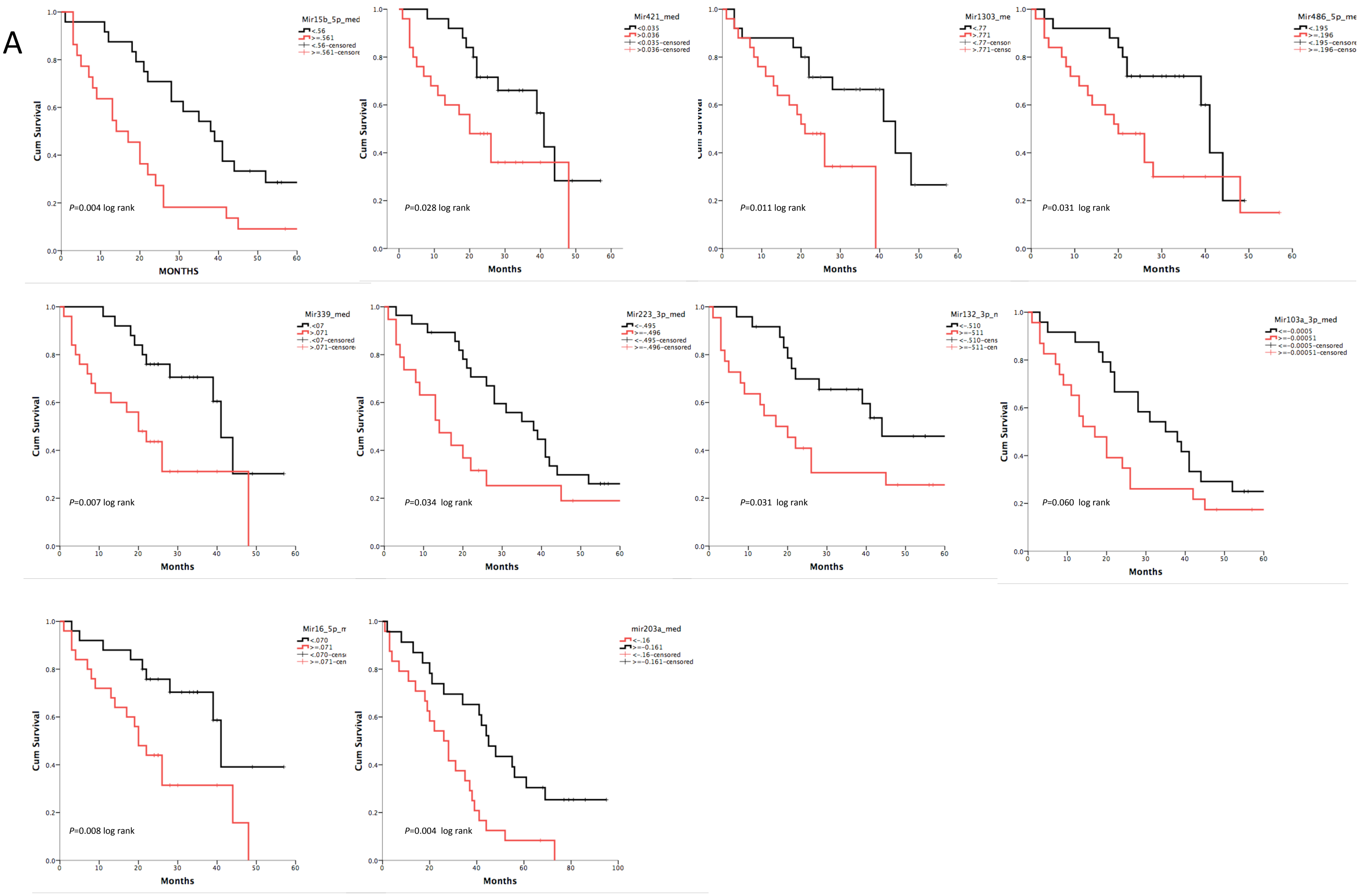

B

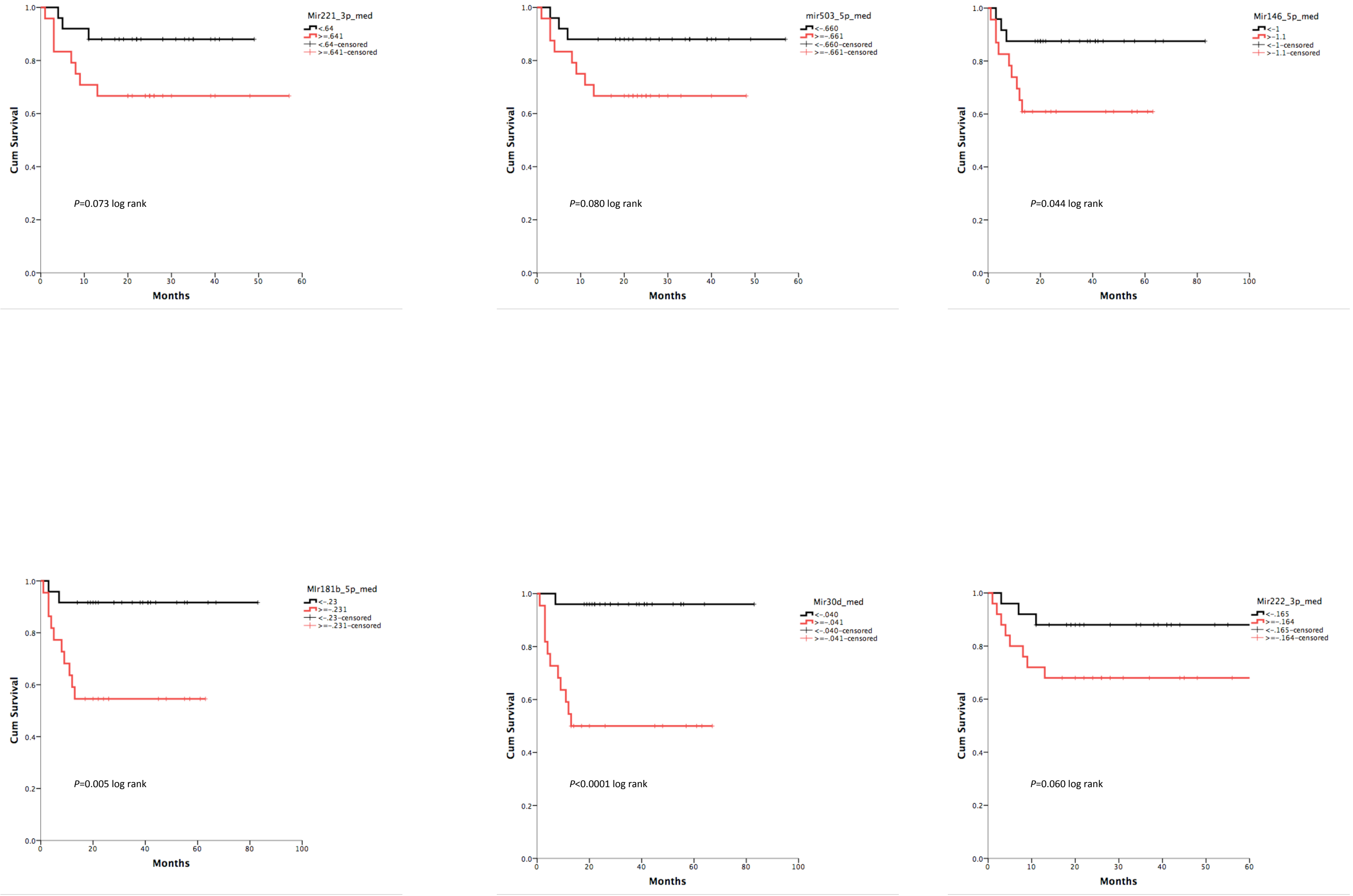
Mir221_3p_med
<.64
>=.641
<.64-censored
>=.641-censored
P=0.073 log rank
Cum Survival
Months
mir503_5p_med
<-.660
>=-.661
<-.660-censored
>=-.661-censored
P=0.080 log rank
Cum Survival
Months
Mir146_5p_med
<-1
>-1.1
<-1-censored
>-1.1-censored
P=0.044 log rank
Cum Survival
Months
MIr181b_5p_med
<-.23
>=-.231
<-.23-censored
>=-.231-censored
P=0.005 log rank
Cum Survival
Months
Mir30d_med
<-.040
>=-.041
<-.040-censored
>=-.041-censored
P<0.0001 log rank
Cum Survival
Months
Mir222_3p_med
<-.165
>=-.164
<-.165-censored
>=-.164-censored
P=0.060 log rank
Cum Survival
Months
