# Supplementary material for: Microenvironment inflammatory infiltrate drives growth speed and outcome of hepatocellular carcinoma: a prospective clinical study
Source: Cell Death Dis. 2017 Aug 24;8(8):e3017–. doi: 10.1038/cddis.2017.395 (PMC5596578; doi:10.1038/cddis.2017.395)
Supplement: Supplementary Table 1 [file cddis2017395x4.docx]

Supplementary Table 1.

Demographic characteristics at enrolment of the patients with Child-Pugh A liver cirrhosis, with HCC diagnosed on surveillance, stratified by growth pattern, who were reported in detail in reference 5.

| **Variable** | **Slow growing**  **HCC**  **n = 98** | **Fast growing HCC**  **n = 34** | **p** |
| --- | --- | --- | --- |
| Male, n (%) | 76 (77.5) | 24 (70.6) |  |
| Median age, y (range) | 66.5 | 70.0 | .700 |
| Etiology, n (%)  HCV  HBV  Alcohol  Dysmetabolic | 60 (61.3)  12 (12.2)  15 (15.3)  11 (11.2) | 15 (44.1)  3 (8.8)  7 (20.6)  9 (26.5) | .123 |
| Performance status, n (%)  0  1 | 93 (94.9)  5 (5.1) | 32 (94.1)  2 (5.9) |  |
| MELD | 9.22±3.9 | 8.73±2.9 | .447 |
| **Biochemistry panel, mean ± SD** |  |  |  |
| Hb (g%) | 13.1±1.7 | 13.2±1.7 | .920 |
| Platelets (x10^3^/mm^3^) | 114275±57747 | 147000±75340 | .023 |
| White blood cells (x10^3^/mm^3^) | 5207.5±2044.4 | 5924.3±2983.9 | .239 |
| Blood glucose (mg/dL) | 114.0±34.9 | 112.9±32.5 | .873 |
| Cholesterol (mg/dL) | 143.3±37.0 | 153.5±53.6 | .378 |
| Blood iron (ng/mL) | 122.4±69.9 | 107.9±46.2 | .287 |
| Ferritin (ng/mL) | 291.3±404.2 | 437.1±663.2 | .362 |
| Albumin (g/dL) | 3.6±0.6 | 3.5±0.7 | .550 |
| CRP ( mg/dL) | 0.5±0.4 | 2.9±1.6 | .036 |
| Creatinine (mg/dL) | 0.9±0.3 | 0.8±0.3 | .231 |
| Bilirubin (mg/dL) | 1.5±2.7 | 1.6±1.9 | .987 |
| INR | 1.2±0.2 | 1.2±0.2 | .138 |
| AST (IU/mL) | 80.2±60.1 | 86.7±79.2 | .690 |
| ALT (IU/mL ) | 65.8±58.2 | 63.6±45.0 | .839 |
| GGT (IU/mL) | 113.6±132.4 | 195.8±271.0 | .133 |
| ALP (IU/mL) | 162.4±137.8 | 164.8±113.2 | .929 |
| Na (mEq/L) | 138.9±3.7 | 138.2±3.7 | .389 |
| AFP (ng/ml) | 105±775 | 1539±5012 | .006 |

|  |  |
| --- | --- |

Abbreviations: HCV, hepatitis C virus; HBV, hepatitis B virus; MELD: Hb, Hemoglobin; CRP: C-reactive protein; INR, International Normalized Ratio; AST, aspartate aminotransferase; ALT, Alanine aminotransferase; GGT, Gamma-glutamyl transferase; ALP, alkaline phosphatase; Na, sodium; AFP, alpha-fetoprotein,
