# Supplementary material for: Microenvironment inflammatory infiltrate drives growth speed and outcome of hepatocellular carcinoma: a prospective clinical study
Source: Cell Death Dis. 2017 Aug 24;8(8):e3017–. doi: 10.1038/cddis.2017.395 (PMC5596578; doi:10.1038/cddis.2017.395)
Supplement: Supplementary Table 3 [file cddis2017395x6.docx]

| Biological significance | miRNA | Pathway and molecular targets |
| --- | --- | --- |
| apoptosis | miR-375 ^(1,2)^; miR-122-5p ^(3,4)^; miR-9-3p e mir-9-5p ^(7,8)^; miR-30a-5p ^(18-20)^; miR-29b-3p ^(16,17)^; miR-144-3p ^(11,12)^; miR-193a-3p ^(9, 10)^; miR-210-3p ^(28)^; miR-513a-5p ^(49)^; miR-29c-3p ^(60)^; miR-26b-5p ^(74)^; let-7g-5p ^(75,76)^; miR-125a-5p ^(78)^; miR-149-5p ^(83,84)^; miR-191-5p ^(85)^; miR-30e-5p ^(89,90)^; let-7a-5p ^(91, 92)^; let-7c-5p e let-7c-3p ^(93-95)^; miR-15b-5p e miR-16-5p ^(100)^; miR-34a-5p ^(119-121)^ | bcl2/1, MCL1 naip5, fgfr2,myb2, caspase-3 |
| cell proliferation | miR-375 ^(1,2)^; miR-9-3p ^(7,8)^; miR-193a-3p ^(9,10)^; miR-144-3p ^(11,12)^; miR-29a-3p ^(13-15)^; miR-29b-3 ^(16,17)^; miR-30a-5p ^(18-20)^; miR-192 -5p ^(21,22)^; miR-218-5p ^(25,26)^; miR-210-3p ^(28)^; miR-296-5p ^(29-32)^; miR-17-5p ^(33)^; miR-17-3p ^(34)^; miR-18a-5p ^(35,36)^; miR-200a-3p e miR-200b-3p e miR-200c-3p ^(52-54)^; miR-9-5p ^(58)^; miR-26b-5p ^(74)^; miR-105-5p ^(79)^; miR-191-5p ^(85)^; let-7a-5p ^(91,92)^; let-7d-5p ^(91)^; let-7c-5p e let-7c-3p ^(92,94)^; miR-421 ^(101-103)^; miR-181b-5p ^(109)^; miR-132-3p ^(28)^; miR-30d-5p ^(110,111)^ | TGF-β/BMP, Notch, IGF1R and PIʒK/Akt - mTOR - signaling Pathways  p120RasGAP NRAS,PRAME, CUL4B, PFKFb2, MAP3K8 Claudin-18 gene targeting c-myc HSP-27 |
| cell cycle regulation and cellular metabolism | miR-30a-5p ^(18-20)^; miR-139-5p ^(43,44)^; miR-125a-5p ^(56,57)^; miR-191-5p ^(85)^; miR-221-3p e miR-222-3p ^(105)^; miR-103a-3p ^(112,113)^; miR-34a-5p ^(119-121)^ | PI3K/AKT signaling  CyclinD2, E2 CDK2, CDK4, and CDK6 COL1A2, c-Myc, p16 TP53 |
| angiogenesis | miR-122-5p ^(3,4)^; miR-193a-3p ^(9, 10)^; miR-30a-5p ^(18-20)^; miR-192 -5p ^(21,22)^; miR-542-3p ^(23, 24)^; miR-210-3p ^(28)^; miR-296-5p ^(29-32)^; miR-17-5p ^(33)^; miR-17-3p ^(34)^; miR-92a-1-5p ^(39,40)^; miR-378a-5p ^(41, 42)^; miR-30c-5p ^(59)^; miR-126-3p ^(28-32)^; miR-195-5p ^(73)^; miR-27b-3p ^(88)^; miR-15b-5p ^(100)^; miR-486 ^(106)^; miR-30d-5p ^(28)^; miR-103a-3p ^(112,113)^; miR-503-5p ^(117,118)^; miR-34a-5p ^(119-121)^ | VEGF, ANGPT2, ANGPTL-1 Dll4, TGF-β, PPARγ EGR1-HOXB9 BCR signaling Insulin/PI3K-AKT Signaling FGF2 and VEGFA |
| metastasis, migration and cell invasiveness | miR-122-5p ^(3,4)^; miR-193a-3p ^(9,10)^; miR-144-3p ^(11,12)^; miR-29b-3p ^(16,17)^; miR-218-5p ^(25,26)^; miR-296-5p ^(29,32)^; miR-18a-5p ^(35,36)^; miR-378a-5p ^(41,42)^; miR-139-5p ^(43,44)^; miR-23a-3p ^(45)^; miR-27a-3p ^(46)^; miR-24-3p ^(47)^; miR-200a-3p e miR-200b-3p e miR-200c-3p ^(52-54)^; miR-181a-5p ^(62,62)^; miR-26b-5p ^(74)^; let-7g-5p ^(75,76)^; miR-125a-5p ^(56,57)^; miR-149-5p ^(83,84)^; miR-191-5p ^(91)^; miR-26a-5p ^(86,87)^; miR-1303 ^(104)^; miR-486-5p ^(106)^; miR-181b-5p ^(109)^; miR-30d-5p ^(28)^; miR-19b-2-5p^(122)^ | VE-cadherin RXRα and ABCA1  p53/p21 Pathway importin-α3 TIMP3 DNAJA2, NME7, PCDH10, RHEBL1, SCN4B, ZDHHC7 NDRG1, EPCAM, HIF1A, HMGB2, MAPK14 |
| inflammation | miR-511-5p ^(5)^; miR-511-3p ^(6)^; miR-378a-5p ^(41,42)^; miR-133a-5p e miR-133a-3p ^(48)^; miR-513a-5p ^(49)^; miR-9-5p ^(58)^; miR-30c-5p ^(59)^; miR-145-5p ^(64,65)^; miR-126-3p ^(28,32)^; let-7e-5p ^(80)^; miR-155-5p ^(96-99)^; miR-146a-5p ^(116)^ | modulation of TGFβ pathway NF-κB Pathway TLR4 signalling interleukins |
| autophagy | miR-30a-5p ^(18-20)^; miR-19b-3p e miR-19a-3p ^(55)^; miR-1303 ^(104)^ | Atg2B, AEG-1, |
| innate immune system | miR-511-5p ^(5)^; miR-511-3p ^(6)^; miR-125b-5p ^(56,57)^; miR-20a-5p ^(66,68)^; miR-203a ^(77)^; miR-187-3p ^(81)^; miR-99b-5p ^(82)^; miR-30e-5p ^(89,90)^; miR-155-5p ^(96-99)^; miR-339-5p ^(107,108)^; miR-223-3p ^(114,115)^; miR-146a-5p ^(116)^ | Akt signaling TLR signaling BMF, ERBB2, ERBB3 Regulation of STAT3 |
| B lymphocyte differentiation and activation | miR-29a-3p ^(16,17)^; miR-125b-5p ^(56,57)^; miR-145-5p ^(64,65)^; miR-155-5p ^(96-99)^ | ADAM17 hedgehog, NF-KB signaling and TGF pathway |
| T lymphocyte differentiation and activation | miR-29a-3p ^(16,17)^; miR-29c-3p ^(60)^; miR-181a-5p ^(61,62)^; miR-30b-5p ^(63)^; miR-142-5p ^(57)^; miR-150-5p ^(68)^; miR-214-3p ^(80)^; miR-21-5p ^(69-71)^; miR-203a ^(77)^; miR-187-3p ^(81)^; miR-99b-5p ^(82)^; miR-30e-5p ^(89,90)^; miR-155-5 ^(96-99)^; miR-339-5p ^(107,108)^; miR-223-3p ^(114,115)^; miR-146a-5p ^(116)^; miR-34a-5p ^(119-121)^ | TCR signaling  BCR signaling TGF-β STAT3 3′-UTR of mRNA of GARP |
| platelet aggregation | miR-19b-1-5p ^(37,38)^ |  |
| drug resistance | miR-193a-3p ^(9,10)^; miR-29b-3p ^(16,17)^ | MCL1, CDK6, c-kit, E2F6, LAMC1 and PPIC and LASP1 COL1A1, and COL4A1 MDM2 |
| epithelial mesenchymal transition | miR-27a-3p ^(46)^;miR-29b-3p ^(16,17)^; miR-200a-3p e miR-200b-3p e miR-200c-3p ^(52-54)^; miR-4717-5p e miR-4717-3p ^(50)^ | E-cadherin, N-cadherin, Twist, and Snail, GFA, ANGPT-L4, PDGF, LOX, and MMP-9, Wnt/b-catenin, Lamin-Integrin, PD-1, PD-L1 |
|  |  |  |
| References |  |  |
| 1) Lian, Sen, et al. "MicroRNA-375 Functions as a Tumor-Suppressor Gene in Gastric Cancer by Targeting Recepteur d’Origine Nantais." International Journal of Molecular Sciences 17.10 (2016): 1633. | | |
| 2) Wang, Xin-Zheng, et al. "Over-expression of microRNA-375 inhibits papillary thyroid carcinoma cell proliferation and induces cell apoptosis by targeting ERBB2." Journal of pharmacological sciences 130.2 (2016): 78-84. | | |
| 3) Lin, Cliff Ji-Fan, et al. "miR-122 targets an anti-apoptotic gene, Bcl-w, in human hepatocellular carcinoma cell lines." Biochemical and biophysical research communications 375.3 (2008): 315-320. | | |
| 4) Xu, Jie, et al. "MicroRNA‐122 suppresses cell proliferation and induces cell apoptosis in hepatocellular carcinoma by directly targeting Wnt/β‐catenin pathway." Liver International 32.5 (2012): 752-760. | | |
| 5) Augello, Claudia, et al. "MicroRNA as potential biomarker in HCV-associated diffuse large B-cell lymphoma." Journal of clinical pathology (2014): jclinpath-2014. | |  |
| 6) Heinsbroek, Sigrid EM, et al. "miR-511-3p, embedded in the macrophage mannose receptor gene, contributes to intestinal inflammation." Mucosal immunology (2015). | | |
| 7) Chen, Yangjing, et al. "Upregulated miR-9-3p promotes cell growth and inhibits apoptosis in medullary thyroid carcinoma by targeting BLCAP."Oncology Research Featuring Preclinical and Clinical Cancer Therapeutics (2016) | | |
| 8) Higashi, T., et al. "miR-9-3p plays a tumour-suppressor role by targeting TAZ (WWTR1) in hepatocellular carcinoma cells." British journal of cancer 113.2 (2015): 252-258. | | |
| 9) Liu, Yongru, et al. "Down-Regulation of MiR-193a-3p Dictates Deterioration of HCC: A Clinical Real-Time qRT-PCR Study." Medical science monitor: international medical journal of experimental and clinical research 21 (2015): 2352 | | |
| 10) Li, Yang, et al. "The miR-193a-3p-regulated ING5 gene activates the DNA damage response pathway and inhibits multi-chemoresistance in bladder cancer." Oncotarget 6.12 (2015): 10195. | | |
| 11) Liu, Fei, et al. "miR-144-3p serves as a tumor suppressor for renal cell carcinoma and inhibits its invasion and metastasis by targeting MAP3K8." Biochemical and Biophysical Research Communications 480.1 (2016): 87-93. | | |
| 12) Huo, Fangyi, et al. "MicroRNA-144-3p inhibits proliferation and induces apoptosis of human salivary adenoid carcinoma cells via targeting of mTOR." Biotechnology letters 38.3 (2016): 409-416. | | |
| 13) Filip, Agata A., et al. "Expression of circulating miRNAs associated with lymphocyte differentiation and activation in CLL—another piece in the puzzle." Annals of Hematology 96.1 (2017): 33-50. | | |
| 14) Zhao, Zhujiang, et al. "Reduced miR-29a-3p expression is linked to the cell proliferation and cell migration in gastric cancer." World journal of surgical oncology 13.1 (2015): 1. | | |
| 15) Wang, Yang, et al. "The role of miRNA-29 family in cancer." European journal of cell biology 92.3 (2013): 123-128. | |  |
| 16) Andrews, Miles C., et al. "Systems analysis identifies miR-29b regulation of invasiveness in melanoma." Molecular Cancer 15.1 (2016): 72. | |  |
| 17) Yan, Bin, et al. "The role of miR-29b in cancer: regulation, function, and signaling." Onco Targets Ther 8 (2015): 539-548. | |  |
| 18) Huang, Wen-Ting, et al. "clinicopathological role of mir-30a-5p in hepatocellular carcinoma tissues and prediction of its function with bioinformatics analysis." OncoTargets and therapy 9 (2016): 5061. | | |
| 19) He, Rongquan, et al. "MiR-30a-5p suppresses cell growth and enhances apoptosis of hepatocellular carcinoma cells via targeting AEG-1." International journal of clinical and experimental pathology 8.12 (2015): 15632. | | |
| 20) Huang, Qing Bo, et al. "Down-regulated miR-30a in clear cell renal cell carcinoma correlated with tumor hematogenous metastasis by targeting angiogenesis-specific DLL4." PloS one 8.6 (2013): e67294. | | |
| 21) Wu, Sherry Y., et al. "A miR-192-EGR1-HOXB9 regulatory network controls the angiogenic switch in cancer." Nature communications 7 (2016). | |  |
| 22) Li, Shujun, et al. "Mir-192 suppresses apoptosis and promotes proliferation in esophageal aquamous cell caicinoma by targeting Bim." International journal of clinical and experimental pathology 8.7 (2015): 8048. | | |
| 23) Wang, Yemin, et al. "p53 is positively regulated by miR-542-3p." Cancer research 74.12 (2014): 3218-3227. | |  |
| 24) He, Ting, et al. "MicroRNA‐542‐3p inhibits tumour angiogenesis by targeting Angiopoietin‐2." The Journal of pathology 232.5 (2014): 499-508. | |  |
| 25) Zhu, Kegan, et al. "Tumor-suppressive miR-218-5p inhibits cancer cell proliferation and migration via EGFR in non-small cell lung cancer." Oncotarget (2016). | |  |
| 26) Taipaleenmäki, Hanna, et al. "Antagonizing miR-218-5p attenuates Wnt signaling and reduces metastatic bone disease of triple negative breast cancer cells." Oncotarget 7.48 (2016): 79032-79046. | | |
| 27) Liu, Yang, et al. "MiR-130a-3p regulates cell migration and invasion via inhibition of Smad4 in gemcitabine resistant hepatoma cells."Journal of Experimental & Clinical Cancer Research 35.1 (2016): 1. | | |
| 28) Guo, Lingling, et al. "MicroRNAs, TGF-β signaling, and the inflammatory microenvironment in cancer." Tumor Biology 37.1 (2016): 115-125. | |  |
| 29) Lee, H., et al. "MicroRNA-296-5p Promotes Invasiveness through Downregulation of Nerve Growth Factor Receptor and Caspase-8." Molecules and cells (2016) | |  |
| 30) Lee, Kuen-Haur, et al. "MicroRNA-296-5p (miR-296-5p) functions as a tumor suppressor in prostate cancer by directly targeting Pin1." Biochimica et Biophysica Acta (BBA)-Molecular Cell Research 1843.9 (2014): 2055-2066. | | |
| 31) Wang, Lina, et al. "Gene and MicroRNA profiling of human induced pluripotent stem cell-derived endothelial cells." Stem Cell Reviews and Reports 11.2 (2015): 219-227 | | |
| 32) Landskroner-Eiger, Shira, Isabelle Moneke, and William C. Sessa. "miRNAs as modulators of angiogenesis." Cold Spring Harbor perspectives in medicine 3.2 (2013): a006643 | | |
| 33) Chen, Chun, et al. "MiR‐17‐5p promotes cancer cell proliferation and tumorigenesis in nasopharyngeal carcinoma by targeting p21." Cancer Medicine 5.12 (2016): 3489-3499. | | |
| 34) Shan, Sze Wan, et al. "Mature miR-17-5p and passenger miR-17-3p induce hepatocellular carcinoma by targeting PTEN, GalNT7 and vimentin in different signal pathways." J Cell Sci 126.6 (2013): 1517-1530. | | |
| 35) Song, Yichen, et al. "MiR-18a regulates the proliferation, migration and invasion of human glioblastoma cell by targeting neogenin." Experimental cell research 324.1 (2014): 54-64. | | |
| 36) Liu, Wan–Hsin, et al. "MicroRNA-18a prevents estrogen receptor-α expression, promoting proliferation of hepatocellular carcinoma cells." Gastroenterology 136.2 (2009): 683-693. | | |
| 37) Kok, M. G. M., et al. "Low miR-19b-1-5p expression in isolated platelets after aspirin use is related to aspirin insensitivity." Circulating microRNAs and other biomarkers for premature atherosclerosis (2015): 43. | | |
| 38) Kok, Maayke Guda Maria. Circulating microRNAs and other biomarkers for premature atherosclerosis. 2015. | |  |
| 39) Singh, Neha, et al. "Endothelium-enriched microRNAs as diagnostic biomarkers for cardiac allograft vasculopathy." The Journal of Heart and Lung Transplantation 34.11 (2015): 1376-1384. | | |
| 40) Bonauer, Angelika, et al. "MicroRNA-92a controls angiogenesis and functional recovery of ischemic tissues in mice." Science 324.5935 (2009): 1710-1713 | |  |
| 41) Krist, Bart, et al. "The Role of miR-378a in Metabolism, Angiogenesis, and Muscle Biology." International journal of endocrinology 2015 (2015). | |  |
| 42) Li, Haoran, et al. "Anti-microRNA-378a enhances wound healing process by upregulating integrin beta-3 and vimentin." Molecular Therapy 22.10 (2014): 1839-1850. | | |
| 43) Chen, Hong, et al. "mir-139-5p regulates proliferation, apoptosis, and cell cycle of uterine leiomyoma cells by targeting TPD52." OncoTargets and therapy 9 (2016): 6151. | | |
| 44) Song, Mingxu, et al. "MiR-139-5p inhibits migration and invasion of colorectal cancer by downregulating AMFR and NOTCH1." Protein & cell 5.11 (2014): 851-861. | |  |
| 45) Wen, Yu-Ching, et al. "By inhibiting snail signaling and miR-23a-3p, osthole suppresses the EMT-mediated metastatic ability in prostate cancer." Oncotarget 6.25 (2015): 21120. | | |
| 46) hao, Nan, et al. "miR-27a-3p suppresses tumor metastasis and VM by down-regulating VE-cadherin expression and inhibiting EMT: an essential role for Twist-1 in HCC." Scientific reports 6 (2016). | | |
| 47) Zhang, Ming-xue, et al. "miR-24-3p Suppresses Malignant Behavior of Lacrimal Adenoid Cystic Carcinoma by Targeting PRKCH to Regulate p53/p21 Pathway."PloS one 11.6 (2016): e0158433. | | |
| 48) Roderburg, Christoph, et al. "miR-133a mediates TGF-β-dependent derepression of collagen synthesis in hepatic stellate cells during liver fibrosis." *Journal of hepatology* 58.4 (2013): 736-742 | | |
| 49) Shin, Sojin, et al. "MicroRNA-513a-5p mediates TNF-α and LPS induced apoptosis via downregulation of X-linked inhibitor of apoptotic protein in endothelial cells." Biochimie 94.6 (2012): 1431-1436 | | |
| 50) Zou, Peng, et al. "ROS generation mediates the anti-cancer effects of WZ35 via activating JNK and ER stress apoptotic pathways in gastric cancer." *Oncotarget* 6.8 (2015): 5860. | | |
| 51) Li, Zhuo-Jian, Pai-Huai Ou-Yang, and Xing-Peng Han. "Profibrotic effect of miR-33a with Akt activation in hepatic stellate cells."*Cellular signalling* 26.1 (2014): 141-148. | | |
| 52) Gibbons, Don L., et al. "Abstract A21: microRNA-200 regulates tumor cell PD-L1 expression to control lung cancer metastasis." Clinical Cancer Research 20.2 Supplement (2014): A21-A21. | | |
| 53) Yuan, Ji‐hang, et al. "The histone deacetylase 4/SP1/microrna‐200a regulatory network contributes to aberrant histone acetylation in hepatocellular carcinoma." Hepatology 54.6 (2011): 2025-2035. | | |
| 54) Korpal, Manav, et al. "The miR-200 family inhibits epithelial-mesenchymal transition and cancer cell migration by direct targeting of E-cadherin transcriptional repressors ZEB1 and ZEB2." Journal of Biological Chemistry 283.22 (2008): 14910-14914. | | |
| 55) Zou, Meijuan, et al. "Autophagy inhibition of hsa-miR-19a-3p/19b-3p by targeting TGF-β R II during TGF-β1-induced fibrogenesis in human cardiac fibroblasts." *Scientific reports* 6 (2016). | | |
| 56) Rückerl, Dominik, et al. "Induction of IL-4Rα–dependent microRNAs identifies PI3K/Akt signaling as essential for IL-4–driven murine macrophage proliferation in vivo." Blood 120.11 (2012): 2307-2316. | | |
| 57) Liu, Juan, et al. "Mechanism of T cell regulation by microRNAs." Cancer biology & medicine 10.3 (2013): 131-137. | |  |
| 58) Girardi, C., et al. "Integration analysis of microRNA and mRNA expression profiles in human peripheral blood lymphocytes cultured in modeled microgravity." BioMed research international 2014 (2014). | | |
| 59) Bay, Ali, and Enes Coskun. "Evaluation of the Plasma Micro RNA Expression Levels in Secondary Hemophagocytic Lymphohistiocytosis." Mediterranean journal of hematology and infectious diseases 5.1 (2013): 2013066. | | |
| 60) Moffett, Howell Franklin. MicroRNAs in normal and malignant lymphocytes. Diss. 2012. | |  |
| 61) Schaffert, Steven A., et al. "mir-181a-1/b-1 modulates tolerance through opposing activities in selection and peripheral T cell function." The Journal of Immunology 195.4 (2015): 1470-1479. | | |
| 62) Wang B, Hsu SH, Majumder S, Kutay H, Huang W, Jacob ST, et al. Tgfbeta-mediated upregulation of hepatic mir-181b promotes hepatocarcinogenesis by targeting timp3. Oncogene. 2010;29:1787–97. | | |
| 63) Naqvi, Afsar Raza, et al. "miR-24, miR-30b and miR-142-3p interfere with antigen processing and presentation by primary macrophages and dendritic cells." Scientific Reports 6 (2016). | | |
| 64) Tan, Lu Ping, et al. "miRNA profiling of B-cell subsets: specific miRNA profile for germinal center B cells with variation between centroblasts and centrocytes." Laboratory Investigation 89.6 (2009): 708-716. | | |
| 65) Marques-Rocha, José Luiz, et al. "Noncoding RNAs, cytokines, and inflammation-related diseases." The FASEB Journal 29.9 (2015): 3595-3611. | |  |
| 66) Zhang, Jian, et al. "A seven-microRNA expression signature predicts survival in hepatocellular carcinoma." PloS one 10.6 (2015): e0128628. | |  |
| 67) Vasilatou, Diamantina, et al. "The role of microRNAs in normal and malignant hematopoiesis." European journal of haematology 84.1 (2010): 1-16. | |  |
| 68) Jethwa, Krishan, et al. "miRNA-mediated immune regulation and immunotherapeutic potential in glioblastoma." Clinical investigation 1.12 (2011): 1637-1650. | |  |
| 69) Rouas, Redouane, et al. "Human natural Treg microRNA signature: role of microRNA‐31 and microRNA‐21 in FOXP3 expression." European journal of immunology 39.6 (2009): 1608-1618. | | |
| 70) Calin GA, Croce CM. MicroRNA signatures in human cancers. Nat Rev Cancer. 2006;6:857–66. | |  |
| 71) Iliopoulos D, Hirsch HA, Struhl K. An epigenetic switch involving nf-kappab, lin28, let-7 microRNA, and il6 links inflammation to cell transformation. Cell. 2009;139:693–706. | | |
| 72) Pfeffer SR, Yang CH, Pfeffer LM. The role of mir-21 in cancer. Drug Deve Res. 2015. doi:10.1002/ddr.21257. | |  |
| 73) Sandrim, Valeria Cristina, et al. "Plasma from pre‐eclamptic patients induces the expression of the anti‐angiogenic miR‐195‐5p in endothelial cells." Journal of cellular and molecular medicine 20.6 (2016): 1198-1200. | | |
| 74) Du, Jing-Yu, et al. "miR-26b inhibits proliferation, migration, invasion and apoptosis induction via the downregulation of 6-phosphofructo-2-kinase/fructose-2, 6-bisphosphatase-3 driven glycolysis in osteosarcoma cells." Oncology reports 33.4 (2015): 1890-1898. | | |
| 75) Shimizu, Satoshi, et al. "The let-7 family of microRNAs inhibits Bcl-xL expression and potentiates sorafenib-induced apoptosis in human hepatocellular carcinoma." Journal of hepatology 52.5 (2010): 698-704. | | |
| 76) Ji, Junfang, et al. "Let-7g targets collagen type I α2 and inhibits cell migration in hepatocellular carcinoma." Journal of hepatology 52.5 (2010): 690-697. | |  |
| 77) Brogaard, Louise, et al. "Late regulation of immune genes and microRNAs in circulating leukocytes in a pig model of influenza A (H1N2) infection." Scientific reports 6 (2016) | | |
| 78) Godlewski, Jakub, et al. "Targeting of the Bmi-1 oncogene/stem cell renewal factor by microRNA-128 inhibits glioma proliferation and self-renewal." Cancer research 68.22 (2008): 9125-9130. | | |
| 79) Shen, Gang, et al. "MicroRNA-105 suppresses cell proliferation and inhibits PI3K/AKT signaling in human hepatocellular carcinoma." Carcinogenesis (2014): bgu208. | |  |
| 80) Paladini, Laura, et al. "Targeting microRNAs as key modulators of tumor immune response." Journal of Experimental & Clinical Cancer Research 35.1 (2016): 103. | |  |
| 81) Squadrito, Mario Leonardo, et al. "MicroRNA-mediated control of macrophages and its implications for cancer." Trends in immunology 34.7 (2013): 350-359. | |  |
| 82) Lindsay, Mark A. "microRNAs and the immune response." Trends in immunology 29.7 (2008): 343-351. | |  |
| 83) Jin, Lu, et al. "Tumor suppressor miR-149-5p is associated with cellular migration, proliferation and apoptosis in renal cell carcinoma." Molecular medicine reports 13.6 (2016): 5386-5392. | | |
| 84) Grieco, Fabio Arturo, et al. "MicroRNAs miR-23a-3p, miR-23b-3p and miR-149-5p Regulate the Expression of Pro-Apoptotic BH3-Only Proteins DP5 and PUMA in Human Pancreatic Beta Cells." Diabetes (2016): db160592. | | |
| 85) Nagpal, Neha, and Ritu Kulshreshtha. "miR-191: an emerging player in disease biology." Frontiers in genetics 5 (2014): 99. | |  |
| 86) Yang, Xin, et al. "MicroRNA‐26a suppresses tumor growth and metastasis of human hepatocellular carcinoma by targeting interleukin‐6‐Stat3 pathway." Hepatology 58.1 (2013): 158-170. | | |
| 87) Kota, Janaiah, et al. "Therapeutic microRNA delivery suppresses tumorigenesis in a murine liver cancer model." Cell 137.6 (2009): 1005-1017 | |  |
| 88) Veliceasa, Dorina, et al. "Therapeutic manipulation of angiogenesis with miR-27b." Vascular cell 7.1 (2015): 6. | |  |
| 89) Yu, Hong WH, Daniel MY Sze, and William Cho. "MicroRNAs involved in anti-tumour immunity." International journal of molecular sciences 14.3 (2013): 5587-5607. | |  |
| 90) Guo, Yan, et al. "MicroRNA-30e Targets BNIP3L to Protect against Aldosterone-induced Podocyte Apoptosis and Mitochondrial Dysfunction." American Journal of Physiology-Renal Physiology (2016): ajprenal-00486. | | |
| 91)Wang, Zifeng, et al. "MYC protein inhibits transcription of the microRNA cluster MC-let-7a-1∼ let-7d via noncanonical E-box." Journal of Biological Chemistry 286.46 (2011): 39703-39714. | | |
| 92) Tsang, Wing Pui, and Tim Tak Kwok. "Let-7a microRNA suppresses therapeutics-induced cancer cell death by targeting caspase-3." Apoptosis 13.10 (2008): 1215-1222 | | |
| 93)Au, Sandy Leung‐Kuen, et al. "Enhancer of zeste homolog 2 epigenetically silences multiple tumor suppressor microRNAs to promote liver cancer metastasis." Hepatology 56.2 (2012): 622-631. | | |
| 94) Zhu, X. M., et al. "Let-7c microRNA expression and clinical significance in hepatocellular carcinoma." Journal of International Medical Research 39.6 (2011): 2323-2329. | | |
| 95) Shah, Yatrik M., et al. "Peroxisome proliferator-activated receptor α regulates a microRNA-mediated signaling cascade responsible for hepatocellular proliferation." Molecular and Cellular Biology 27.12 (2007): 4238-4247 | | |
| 96) Ha, Tai-You. "The role of microRNAs in regulatory T cells and in the immune response." Immune network 11.1 (2011): 11-41. | |  |
| 97) Tili E, Croce CM,Michaille JJ.Mir-155: on the crosstalk between inflammation and cancer. Int Rev Immunol. 2009;28:264–84. | |  |
| 98) Jurkovicova D, Magyerkova M, Kulcsar L, Krivjanska M, Krivjansky V, Gibadulinova A, et al. Mir-155 as a diagnostic and prognostic marker in hematological and solid malignancies. Neoplasma. 2014;61:241–51. | | |
| 99) Tili E, Michaille JJ, Cimino A, Costinean S, Dumitru CD, Adair B, et al. Modulation of mir-155 and mir-125b levels following lipopolysaccharide/tnf-alpha stimulation and their possible roles in regulating the response to endotoxin shock. J Immunol. 2007;179:5082–9. | | |
| 100) Tsao, Simon Chang-Hao, et al. "The role of circulating microRNA in hepatocellular carcinoma." *FRONTIERS IN BIOSCIENCE-LANDMARK* 20 (2015): 78-104. | |  |
| 101) Li, Yu, et al. "Downregulation of tumor suppressor menin by miR-421 promotes proliferation and migration of neuroblastoma." *Tumor Biology* 35.10 (2014): 10011-10017. | | |
| 102) Hadjimichael, Christiana, et al. "MicroRNAs for Fine-Tuning of Mouse Embryonic Stem Cell Fate Decision through Regulation of TGF-β Signaling." Stem cell reports 6.3 (2016): 292-301. | | |
| 103) Liu, Haiyan, et al. "Correlation between microRNA-421 expression level and prognosis of gastric cancer." International journal of clinical and experimental pathology 8.11 (2015): 15128 | | |
| 104) Au, Kin Yi, et al. "MiR-1303 regulates mycobacteria induced autophagy by targeting Atg2B." PloS one 11.1 (2016): e0146770. | |  |
| 105) Matsuzaki, Juntaro, and Hidekazu Suzuki. "Role of MicroRNAs-221/222 in digestive systems." *Journal of clinical medicine* 4.8 (2015): 1566-1577 | |  |
| 106) Liu, Chengxia, et al. "miR-486-5p attenuates tumor growth and lymphangiogenesis by targeting neuropilin-2 in colorectal carcinoma." *OncoTargets and therapy* 9 (2016): 2865. | | |
| 107) Wang, Yi-Lin, et al. "Effects of miR-339-5p on invasion and prognosis of hepatocellular carcinoma." Clinics and research in hepatology and gastroenterology 40.1 (2016): 51-56. | | |
| 108) Zhou, Chang, Yenxia Lu, and Xuenong Li. "miR‑339‑3p inhibits proliferation and metastasis of colorectal cancer." Oncology letters 10.5 (2015): 2842-2848. | |  |
| 109) Wang, Bo, et al. "TGFβ-mediated upregulation of hepatic miR-181b promotes hepatocarcinogenesis by targeting TIMP3." Oncogene 29.12 (2010): 1787-1797. | |  |
| 110) Bridge, Gemma, et al. "The microRNA-30 family targets DLL4 to modulate endothelial cell behavior during angiogenesis." Blood 120.25 (2012): 5063-5072. | |  |
| 111) Yao, Jian, et al. "MicroRNA‐30d promotes tumor invasion and metastasis by targeting Galphai2 in hepatocellular carcinoma." Hepatology 51.3 (2010): 846-856. | |  |
| 112) Trajkovski, Mirko, et al. "MicroRNAs 103 and 107 regulate insulin sensitivity." Nature 474.7353 (2011): 649-653. | |  |
| 113) Liao, Yalin, and Bo Lönnerdal. "Global microRNA characterization reveals that miR-103 is involved in IGF-1 stimulated mouse intestinal cell proliferation." PloS one 5.9 (2010): e12976 | | |
| 114) Jia, Cheng You, et al. "MiR-223 suppresses cell proliferation by targeting IGF-1R." PloS one 6.11 (2011): e27008. | |  |
| 115) Johnnidis, Jonathan B., et al. "Regulation of progenitor cell proliferation and granulocyte function by microRNA-223." Nature 451.7182 (2008): 1125-1129 | |  |
| 116) Chen, Yuhan, et al. "MicroRNA-146a-5p Negatively Regulates Pro-Inflammatory Cytokine Secretion and Cell Activation in Lipopolysaccharide Stimulated Human Hepatic Stellate Cells through Inhibition of Toll-Like Receptor 4 Signaling Pathways. " *International Journal of Molecular Sciences* 17.7 (2016): 1076.**.** | | |
| 117) Kwon, Deug-Nam, Byung-Soo Chang, and Jin-Hoi Kim. "MicroRNA dysregulation in liver and pancreas of CMP-Neu5Ac hydroxylase null mice disrupts insulin/PI3K-AKT signaling." BioMed research international 2014 (2014). | | |
| 118) Zhou, Bisheng, et al. "MicroRNA-503 targets FGF2 and VEGFA and inhibits tumor angiogenesis and growth." Cancer letters 333.2 (2013): 159-169. | |  |
| 119) Cortez, Maria Angelica, et al. "PDL1 regulation by p53 via miR-34." Journal of the National Cancer Institute 108.1 (2016): djv303. | |  |
| 120) Li, Na, et al. "miR-34a inhibits migration and invasion by down-regulation of c-Met expression in human hepatocellular carcinoma cells." Cancer letters 275.1 (2009): 44-53 | | |
| 121) Yang, Pengyuan, et al. "TGF-β-miR-34a-CCL22 signaling-induced Treg cell recruitment promotes venous metastases of HBV-positive hepatocellular carcinoma." Cancer cell 22.3 (2012): 291-303. | | |
| 122) Hang, Zi-Zhen, et al. "Analysis of plasma MicroRNAs to identifying early diagnostic molecule for gastric cancer." International journal of clinical and experimental medicine 8.3 (2015): 3700. | | |
